# Severe pediatric hypocalcemia in Vietnam: etiologic profile, clinical outcomes and risk factors in 246 cases

**DOI:** 10.3389/fendo.2026.1701713

**Published:** 2026-02-02

**Authors:** Thanh T. Nguyen, Giang T.K. Dang, Phuong Thao Bui, Mai Kieu Anh, Pham Thi Thuy Hoa, Dongryeol Ryu, Vu Chi Dung

**Affiliations:** 1 Center for Endocrinology, Metabolism, Genetic/Genomics and Molecular Therapy, Vietnam National Children’s Hospital, Hanoi, Vietnam; 2 Department of Biomedical Science and Engineering, Gwangju Institute of Science and Technology (GIST), Gwangju, Republic of Korea; 3 Neonatology Department, Vinmec Times City International Hospital, Hanoi, Vietnam; 4 Center for Pediatrics, Bach Mai Hospital, Hanoi, Vietnam; 5 Department of Pediatrics, University of Medicine and Pharmacy - Vietnam National University, Hanoi, Vietnam

**Keywords:** DiGeorge syndrome, hypocalcemia, hypoparathyroidism, pediatrics, seizures, vitamin D deficiency, hypocalcemic cardiomyopathy

## Abstract

**Background:**

Severe hypocalcemia in children can precipitate life-threatening neurologic and cardiovascular events, including seizures and cardiac dysfunction. Etiologies range from nutritional deficiency to genetic syndromes, yet local epidemiologic and clinical data remain limited, particularly in Southeast Asia.

**Methods:**

We retrospectively reviewed 246 children (newborns to 18 years) admitted with severe hypocalcemia at the Vietnam National Children’s Hospital (2018 - 2024), the leading tertiary center in Northern Vietnam. Demographic, clinical, and comprehensive biochemical data were collected. Descriptive, bivariate, and multivariable logistic regression analyses identified independent predictors of major clinical outcomes.

**Results:**

Among 246 children, infants and young children predominated (70.7%; median age 67 days). Seizures occurred in 79.2%, while vitamin D deficiency (67.1%) and hypoparathyroidism (28.0%) were the leading causes of severe hypocalcemia. The mean ionized calcium level was profoundly low (0.66 ± 0.13 mmol/L). Patients with vitamin D deficiency had markedly low 25(OH)D levels (mean 18.4 nmol/L) and high PTH (mean 261.7 pg/mL), consistent with secondary hyperparathyroidism. Conversely, those with hypoparathyroidism showed low PTH levels (22.2 pg/mL) despite significant hypocalcemia. Children with DiGeorge syndrome had even lower PTH levels (6.3 pg/mL) and preserved phosphate. In multivariate analyses, lower ionized calcium and lower 25-hydroxyvitamin D levels were independent predictors of seizures. Cardiac complications (cardiogenic shock and/or acute heart failure) occurred in 5.7% and were associated with more severe hypocalcemia.

**Conclusion:**

Severe pediatric hypocalcemia in Vietnam predominantly affects infants and young children and is largely attributable to preventable vitamin D deficiency. While vitamin D deficiency is globally recognized, this study provides novel region-specific insight into the high frequency of symptomatic presentations, including seizures and cardiac events, in a tropical setting with presumed adequate sunlight exposure. It also emphasizes distinct biochemical phenotypes that allow early etiological stratification. These findings reinforce the urgency of proactive vitamin D supplementation policies and biochemical screening protocols tailored for high-risk populations, particularly in resource-limited settings.

## 1 Introduction

Hypocalcemia, defined by a subnormal concentration of serum calcium, is a common electrolyte disturbance in the pediatric population (1, 2). The physiologically active ionized form of calcium is integral to numerous vital processes, including neuromuscular excitability, cardiac contractility and rhythm, blood coagulation, and bone mineralization (3). Accordingly, disturbances in calcium balance may precipitate manifestations ranging from mild paresthesia to life-threatening seizures, laryngospasm, or arrhythmias (3, 4). Reported prevalence and clinical impact vary by age group, geography, and nutritional status (5, 6).

**Figure 1:**
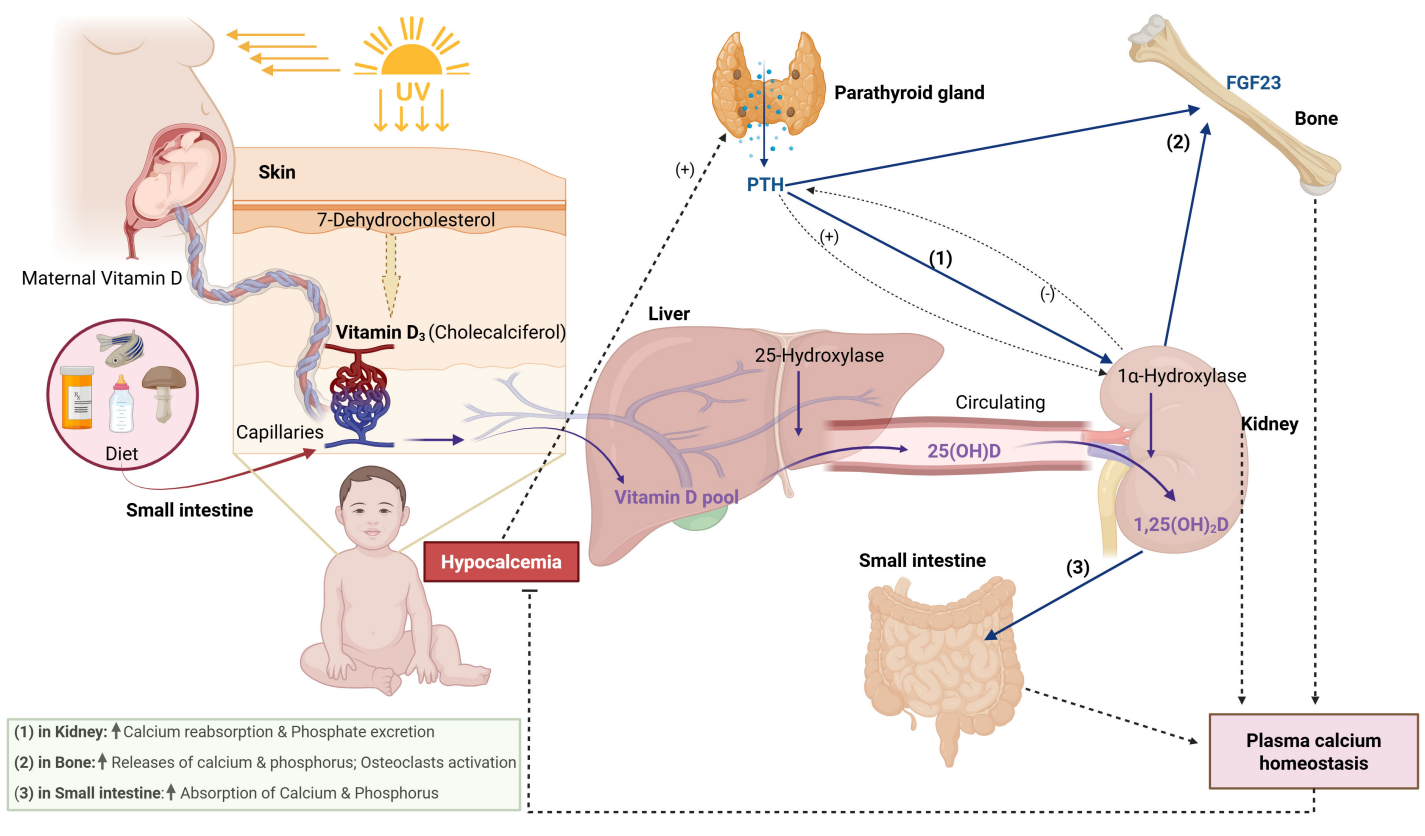
Regulation of calcium and phosphate homeostasis by vitamin D and parathyroid hormone. Schematic representation of the metabolic pathway of Vitamin D and its interplay with PTH in maintaining calcium and phosphate balance. Vitamin D, obtained from dietary sources or synthesized in the skin upon UV light exposure, is metabolized in the liver to 25(OH)D and subsequently in the kidney to its active form, 1,25(OH)2D. This activation is stimulated by PTH, which is secreted by the parathyroid glands in response to low plasma calcium. 1,25(OH)2D and PTH act on target organs to restore homeostasis: (1) In the kidneys, PTH promotes calcium reabsorption and phosphate excretion, while 1,25(OH)2D supports calcium reabsorption; (2) In bone, both hormones synergistically promote calcium and phosphorus release via osteoclast activation; and (3) In the small intestine, 1,25(OH)2D enhances the absorption of calcium and phosphorus. Additionally, FGF23 produced by bone provides negative feedback by inhibiting renal 1α-hydroxylase. Disruptions in this balanced pathway can lead to hypocalcemia.

Calcium homeostasis is regulated by an axis linking parathyroid hormone (PTH), vitamin D metabolites, their target organs (intestines, kidneys, and bone) - and - more recently recognized - fibroblast growth factor 23 (FGF23) (7, 8). As summarized in **Figure 1**, vitamin D_3_ (cholecalciferol) from cutaneous synthesis or diet is converted in the liver to 25-hydroxyvitamin D (25(OH)D) and then in the kidney to the active 1,25-dihydroxyvitamin D (1,25(OH)_2_D) under PTH stimulation. Together with direct PTH actions, 1,25(OH)_2_D increases intestinal calcium absorption, renal calcium reabsorption, and mobilization of calcium from bone, while FGF23 counterbalances by downregulating renal 1α-hydroxylase activity. Disruption at any node of this axis may produce hypocalcemia (9, 10).

Etiologies in childhood cluster into three broad categories: (i) vitamin D-related disorders - from simple nutritional deficiency to inborn errors of vitamin D metabolism or receptor signaling (11); (ii) hypoparathyroidism (hypoPTH) states - congenital (e.g., 22q11.2 deletion) or acquired (postsurgical, autoimmune) (1); and (iii) miscellaneous causes such as neonatal transient hypoparathyroidism, high phosphate load, hypomagnesemia, renal failure, critical illness, and certain medications (1). Neonatal hypocalcemia presents with distinct early-onset forms, often associated with prematurity, perinatal asphyxia, and maternal diabetes, and late-onset forms, linked to high phosphate intake or transient PTH insufficiency (12). Other causes include hypomagnesemia, hyperphosphatemia from renal failure or tissue breakdown, critical illness, and certain medications. Seizures are a particularly prominent and alarming manifestation, especially in neonates and young infants, and may be the sole presenting sign (13). Cardiac manifestations can include hypotension, impaired myocardial contractility, and electrocardiographic changes such as prolongation of the QTc interval, which can precipitate life-threatening arrhythmias (14). The multifaceted etiologies necessitate a comprehensive diagnostic evaluation, as management strategies are highly dependent on the underlying cause.

Although the general pathophysiology of pediatric hypocalcemia is well-established, there remains a significant gap in the literature regarding the clinical and biochemical profiles of children with severe hypocalcemia in Southeast Asia. Notably, vitamin D deficiency is prevalent even in sun-rich regions. In Vietnam, studies report a prevalence of 30–50% among infants and young children (15–17). Similar patterns are observed in other low- and middle-income countries, where limited sun exposure, exclusive breastfeeding without supplementation, and poor dietary intake contribute to deficiency rates exceeding 50% in some populations (18, 19). This study was designed to address this gap by comprehensively characterizing the clinical presentations and laboratory features of a large cohort of pediatric patients with severe hypocalcemia at a single tertiary care center in Vietnam. The primary aims were to detail the spectrum of severe hypocalcemia in this population, identify independent risk factors for major complications such as seizures and cardiac involvement, and elucidate the biochemical patterns associated with different etiologies, thereby providing valuable insights to inform and improve clinical practice. Our findings, drawn from one of the largest reported cohorts of severe pediatric hypocalcemia to date, carry implications not only for Vietnam but for many regions facing similar nutritional risks.

## 2 Methods

### 2.1 Study design and participants

**Figure 2:**
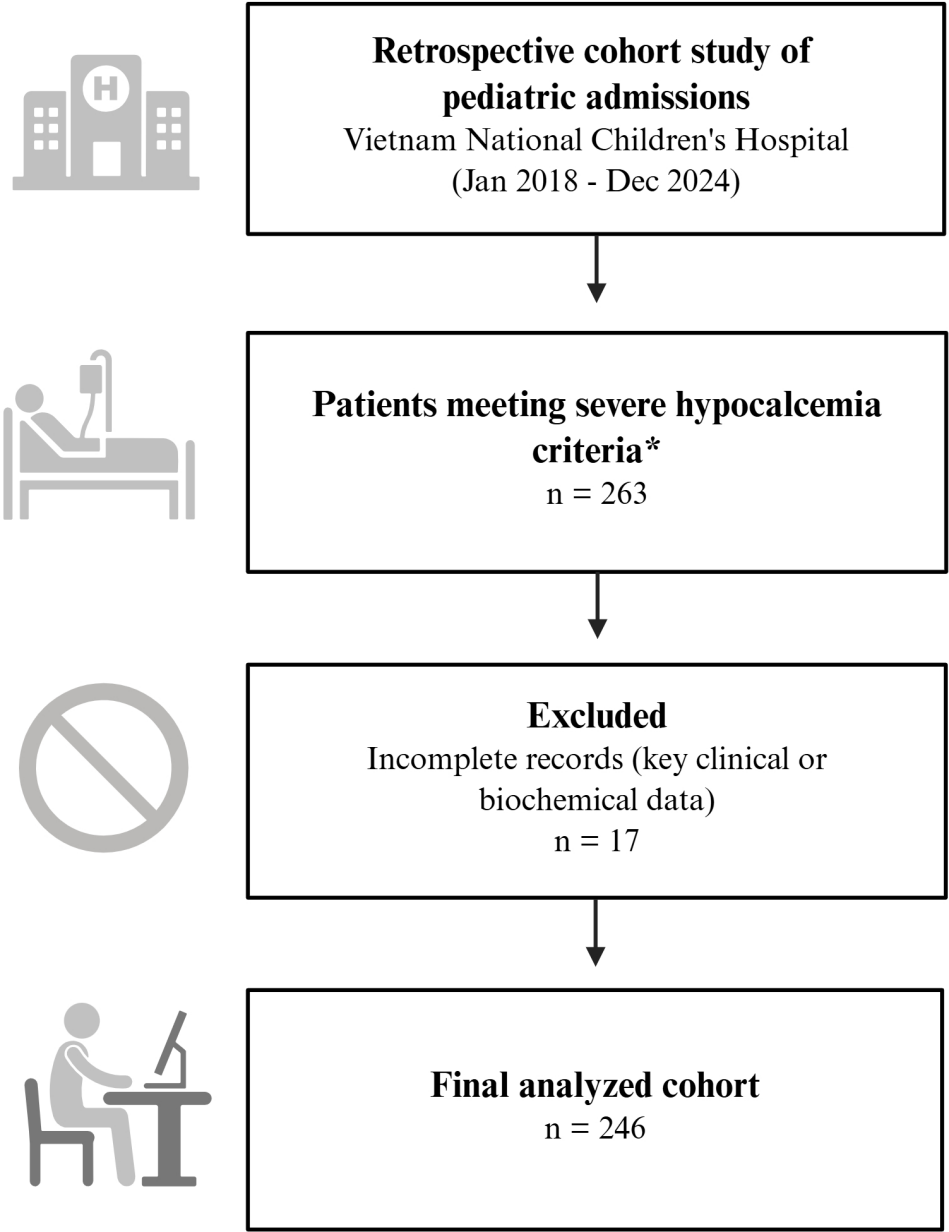
Flowchart of study population selection. The retrospective cohort was established from pediatric admissions at Vietnam National Children's Hospital between 2018 and 2024. Out of 263 patients initially identified, 17 were excluded due to incomplete records, resulting in a final cohort of 246 patients. *Severe hypocalcemia was defined as ionized calcium ≤ 1 mmol/L (4 mg/dL), protein-corrected calcium < 1.75 mmol/L (< 7 mg/dL), or any hypocalcemia presenting with severe symptoms (e.g., seizures, tetany, laryngospasm, or prolonged QTc).

This was a retrospective, cross-sectional study conducted at the Vietnam National Children’s Hospital, a tertiary care pediatric center. The medical records of children aged 0–18 years who were admitted with severe hypocalcemia between January 2018 and December 2024 were reviewed. Inclusion was based on a documented diagnosis of severe hypocalcemia as defined below. Patients with incomplete medical records for key variables essential for the analysis were excluded (**Figure 2**).

The primary aims of the study were:

-To characterize the clinical presentation and biochemical profiles of children with severe hypocalcemia.
-To identify the most common etiologies stratified by age and laboratory features.
-To determine independent risk factors for severe complications (seizures, cardiac events).
-To evaluate the diagnostic utility of calcium-regulating biomarkers (PTH, 25(OH)D) across etiological groups.

### 2.2 Ethical considerations

This study involved a retrospective review of existing, fully anonymized medical records and posed no more than minimal risk to participants. In accordance with the regulations of Vietnam National Children’s Hospital and national guidelines on biomedical research ethics, such studies are exempt from formal Institutional Review Board (IRB) review. The hospital’s Ethics Committee therefore confirmed that the project met exemption criteria and waived the requirement for informed consent (Ref. No. 2075/BVNTWVNCSKTE).

All patient identifiers were removed before data extraction; each record was assigned a unique study code. The de-identified dataset was stored on an encrypted, password-protected server accessible only to the study team. The study complied with the principles of the Declaration of Helsinki and relevant local data-protection laws.

### 2.3 Data collection

Using a standardized case-report form, we extracted (1) demographics (age in days, sex, birthweight, gestational age); (2) clinical data (presenting symptoms, underlying diagnoses, length of stay, ECG/EEG (Electrocardiogram/Electroencephalogram) findings, overt cardiac signs); and (3) biochemical parameters (Protein-corrected Ca (pcCa), ionized Ca, phosphorus, magnesium, albumin, total protein, ALP (Alkaline Phosphatase), 25-hydroxyvitamin D (25(OH)D), intact PTH, and glucose). Samples were collected on admission and were not uniformly fasting. Pre-admission vitamin D/calcium supplementation was recorded when available (yes/no/unknown); dosing details were inconsistently documented and not analyzed quantitatively.

### 2.4 Laboratory measurement

Venous blood (approximately 2–3 mL) was drawn into serum-separator tubes as part of routine clinical care; no additional sampling was performed for the study. After centrifugation, serum biochemistry was analyzed in the central laboratory of Vietnam National Children’s Hospital.

Total calcium, inorganic phosphate, magnesium, albumin, glucose and ALP were measured on an AU5800 analyzer (Beckman Coulter, Brea, CA, USA) using the manufacturer’s photometric methods and reagents. Serum 25(OH)D was quantified by a competitive electrochemiluminescence binding assay (Elecsys Vitamin D total III, Roche Diagnostics) on a cobas pro e 801 immunoassay module; this assay is traceable to an ID-LC-MS/MS reference measurement procedure and standardized against NIST SRM 2972. Intact PTH was measured by a second-generation sandwich electrochemiluminescence immunoassay (Elecsys PTH, Roche Diagnostics) on the same e 801 module, standardized against the WHO International Standard NIBSC 95/646. All assays were calibrated according to the manufacturers’ instructions, and the laboratory performed regular internal quality control and participated in external quality-assessment schemes throughout the study period.

Maternal vitamin D assessment: Maternal serum 25(OH)D was measured only when mothers provided consent for additional testing. Maternal vitamin D status was categorized using the same cutoffs as for children (definitions stated below). Because maternal testing was not systematic, analyses involving maternal 25(OH)D were considered descriptive.

### 2.5 Operational definitions

The following definitions and classifications were used for analysis:

- Severe hypocalcemia: Severe hypocalcemia was defined as either (a) ionized Ca ≤ 1 mmol/L (≤ 4 mg/dL) or protein-corrected Ca < 1.75 mmol/L (< 7 mg/dL), or (b) any hypocalcemia with ≥ 1 severe symptom (carpopedal spasm, laryngo/bronchospasm, seizure, QTc prolongation). Protein-corrected Ca was calculated as pcCa (mmol/L) = total Ca + 0.02 × (40 – albumin [g/L]) (20, 21).
- Neonatal hypocalcemia is defined by specific serum calcium thresholds that differ for term and preterm babies. Term neonates – total serum calcium < 8 mg/dL (approximately 2.0 mmol/L). Preterm neonates – total serum calcium < 7 mg/dL (approximately 1.75 mmol/L) (22). In this study, inclusion was based on the study definition of severe hypocalcemia (pcCa <1.75 mmol/L and/or iCa ≤1.0 mmol/L, or hypocalcemia with severe symptoms), which was applied consistently across all age groups, including neonates.
- Age groups: For analysis, patients were categorized by age into three groups: Neonates (1–28 days), Infants and young children (>28 days – 24 months), and Children (>24 months).
- Vitamin D status: Serum 25(OH)D levels were categorized based on established pediatric guidelines. Severe vitamin D deficiency was defined as <12.5 nmol/L; moderate deficiency as 12.5–29 nmol/L; and mild deficiency as 30–49 nmol/L (23). For etiological categorization in this study, vitamin D deficiency was defined as 25(OH)D <50 nmol/L. Levels 50–75 nmol/L were considered insufficient and were not classified as deficiency for etiological grouping. This approach was chosen to focus on clinically relevant deficiency associated with hypocalcemia and secondary hyperparathyroidism (24–26).
- Cardiac events (cardiac complications): Cardiac events were defined as cardiogenic shock and/or acute heart failure, and all cases meeting these diagnoses had transthoracic echocardiography (TTE) performed during the hypocalcemic episode to assess left ventricular systolic function. Acute heart failure was defined as new-onset or worsening symptoms/signs of heart failure requiring urgent therapy. Reduced left ventricular systolic function was defined as LVEF <55% on TTE (27). Cardiogenic shock was defined as clinical shock with signs of hypoperfusion requiring vasoactive/inotropic support, accompanied by evidence of cardiac dysfunction on TTE (including reduced LVEF) (27, 28). Arrhythmias and QTc prolongation were recorded separately as ECG abnormalities and were not included in the above definition of cardiac events.
- Congenital heart disease (CHD) was defined as any congenital structural cardiac anomaly identified on transthoracic echocardiography performed during admission.
- QT interval and QTc. QT was measured on standard 12-lead ECGs (25 mm/s, 10 mm/mV) from QRS onset to T-wave end in the lead with the clearest T-wave termination. QTc was calculated using Bazett’s formula (QTc = QT/√RR; QT and RR in seconds). QTc prolongation was defined as QTc > age-specific 95th percentile based on pediatric normative charts (28), with thresholds approximately ranging from approximately 478 ms in the first days of life to approximately 446 ms in adolescents. ECGs were interpreted by pediatric cardiologists as part of routine clinical care.
- Hypoparathyroidism: A condition characterized by low or inappropriately normal serum PTH levels in the setting of hypocalcemia, typically accompanied by hyperphosphatemia.
- DiGeorge syndrome (22q11.2 deletion syndrome): The diagnosis is based on clinical features (cardiac anomalies, dysmorphic facial features, immunodeficiency) and confirmed by genetic testing. Hypocalcemia in these patients is due to congenital hypoplasia or aplasia of the parathyroid glands.
- Etiological groups: Etiology was classified into six mutually exclusive groups: (i) Isolated vitamin D deficiency (VDD), (ii) DiGeorge without VDD (DiGeorge), (iii) DiGeorge + VDD, (iv) isolated hypoparathyroidism (HypoPTH), (v) hypoparathyroidism + VDD (HypoPTH + VDD), and (vi) others/unknown.

### 2.6 Statistical analysis

All analyses were performed with IBM SPSS Statistics 26.0 (IBM Corp., Armonk, NY, USA).

Descriptive statistics. Categorical variables are presented as counts and percentages. Continuous variables were assessed for normality using the Shapiro–Wilk test. Normally distributed data are reported as mean ± standard deviation (SD); non-normally distributed data as median with interquartile range (IQR). For multigroup comparisons, summary measures are presented as mean or median for clarity.

Group comparisons. Categorical data were compared with the χ² test or, when ≥25% of expected cell counts were <5, Fisher’s exact test. For continuous data, two-group comparisons used the independent-samples t-test (normal distribution) or the Mann–Whitney U test (non-normal distribution). Comparisons across ≥3 groups employed one-way ANOVA or the Kruskal-Wallis test, respectively. Monotonic associations between biochemical indices were assessed with Spearman’s rank correlation coefficient (ρ).

Multivariable modelling. Variables with p < 0.10 in bivariate analysis - or of known biological relevance - were entered (enter method) into binary logistic regression models to identify independent predictors of seizures and cardiac involvement. Multicollinearity was assessed with the variance inflation factor (VIF); variables with VIF > 5 were excluded. Model calibration and discrimination were evaluated using the Hosmer–Lemeshow goodness-of-fit test, Nagelkerke R², and the area under the receiver-operating-characteristic curve (AUC, 95% CI). For continuous outcomes (ionized calcium concentration, length of stay), multiple linear regression was applied after verifying homoscedasticity; standardized β-coefficients and 95% CIs are reported.

Missing data. Missing values were <5% per variable and were handled by list-wise deletion.

Two-tailed p-values <0.05 were considered statistically significant. No adjustment for multiple comparisons was applied; therefore, inferential results should be interpreted as exploratory.

## 3 Results

### 3.1 Cohort demographics and clinical characteristics

A total of 246 pediatric patients with severe hypocalcemia were included in the study. The baseline demographic and clinical features of the cohort are summarized in **Table 1**. The population was predominantly male (70.7%) and largely composed of infants and young children (70.7%), with a median age at presentation of 67 days (IQR 49.2-146). A substantial proportion of patients (34.1%) were born preterm.

**Table 1:** Baseline demographics and clinical characteristics of the study cohort (N = 246).

Clinical characteristics		Frequency (N)	Percent (%)
Gender	Male	174	70.7
	Female	72	29.3
Age group	Neonates (1day - 28 days)	28	11.4
	Infants and young children (>28 days - 24 months)	174	70.7
	Children (>24 months)	44	17.9
Gestation history	Pre-term	84	34.1
	Full-term	162	65.9
Clinical symptoms	Seizure	195	79.2
	Irritability	89	36.2
	Poor feeding	28	11.4
	Fever	25	10.2
	Lethargy	21	8.5
	Drowsiness	18	7.3
	Overt cardiac signs (cardiogenic shock, heart failure)	14	5.7
	Tetanic spasm	6	2.4
Etiology and co-morbidity	DiGeorge syndrome (22q11.2 del)	27	10.9
	Hypoparathyroidism	69	28.0
	Congenital heart diseases	61	24.8
	Vitamin D deficiency	165	67.1
Abnormal ECG (prolong QTc)		20	8.1
Abnormal EEG (epileptiform)		14	5.7
		Median (IQR)	
Age at presentation (days)		67 (49.20 – 146)	
Hospital stays duration (days)		6.0 (4.40 – 10)	
Birth weight (kg)		2.92 (2.18 – 3.18)	
Body weight (kg)		4.56 (3.70 – 6.84)	

del, deletion; ECG, electrocardiogram; EEG, electroencephalogram; IQR, interquartile range; QTc, corrected QT interval; percentages may exceed 100 % because patients could have >1 condition.

The most frequent clinical presentation was seizures, reported in 195 patients (79.2%), followed by irritability (36.2%). The leading etiologies were nutritional vitamin D deficiency (67.1%) and hypoparathyroidism (28.0%). Notably, 24.8% of patients had underlying congenital heart diseases, reflecting a predisposition of such infants and young children to develop hypocalcemia. The median duration of hospital stay was 6.0 days (IQR 4.4-10), indicating that most children required nearly a week of inpatient management.

### 3.2 Baseline biochemical profile

Baseline laboratory values at presentation (**Table 2**) confirmed markedly low calcium levels across the cohort. The mean ionized calcium was 0.66 ± 0.13 mmol/L, with mean total calcium 1.37 ± 0.24 mmol/L, underscoring the severity of hypocalcemia in this referral population. The median serum 25(OH)D concentration was 24.3 nmol/L (IQR 13.8 - 55.6), with over two-thirds of patients falling into vitamin D-deficient ranges. PTH levels were highly variable (median 185 pg/mL, IQR 39.8 - 313.6). Alkaline phosphatase (ALP), a marker of bone turnover, was often elevated (overall median ~610 U/L), especially in the context of vitamin D-related bone mineralization defects, whereas serum phosphate and magnesium levels varied according to the underlying cause. Additionally, maternal serum 25(OH)D was available for a subset of mother-infant dyads (n = 20). Among these, 90% (18/20) of those mothers had vitamin D insufficient (42.37 nmol/L). Maternal 25(OH)D data were unavailable for the remaining cases because maternal testing required additional consent and was not routinely performed.

**Table 2:** Baseline laboratory parameters of the study cohort (N = 246)^a^.

Parameter	Unit	All patients (N = 246)
pcCa^b^	mmol/L	1.37 ± 0.24
Ionized Ca^b^	mmol/L	0.66 ± 0.13
Protein	g/L	58.9 (54.3 – 68.0)
Albumin^b^	g/L	37.57 ± 4.81
Phosphorus (P)^b^	mmol/L	2.38 ± 0.72
Magnesium (Mg)	mmol/L	0.73 (0.65 – 0.79)
ALP	U/L	610.1 (298.02 – 904.40)
Vitamin D	nmol/L	24.26 (13.75 – 55.57)
PTH	pg/mL	185.00 (39.80 – 313.60)
Glucose	mmol/L	5.0 (4.50 – 5.70)
Maternal Vitamin D (n=20)	nmol/L	42.37 (34.88 – 54.57)

ALP, alkaline phosphatase; pcCa, protein-corrected calcium; PTH, parathyroid hormone.

a Reference ranges for each parameter by age group are provided in **Supplementary Table S1**.

b Variable has normal distribution; values are presented as mean ± SD or median (IQR) as appropriate.

Given that our cohort included both PTH-deficient states (hypoparathyroidism and DiGeorge syndrome) and PTH-sufficient states with secondary hyperparathyroidism (predominantly vitamin D deficiency), we performed Spearman correlation analyses after stratifying patients by PTH status (**Table 3**). In both strata, total calcium correlated strongly with ionized calcium (ρ=0.825–0.853; p<0.001), supporting internal consistency of calcium measurements. In the normal/high-PTH stratum, PTH correlated positively with ALP (ρ=0.333; p<0.001) and ALP correlated inversely with phosphate (ρ=-0.314; p<0.001), consistent with increased bone turnover and phosphaturia in secondary hyperparathyroidism. In the low-PTH stratum, correlations involving PTH were attenuated, while phosphate showed an inverse association with total calcium (ρ=-0.314; p=0.022) and 25(OH)D was inversely correlated with ionized calcium (ρ=-0.262; p=0.038). Overall, these stratified analyses avoid conflating opposing mechanisms and provide a clearer physiologic interpretation of biomarker interrelationships within each PTH-defined subgroup.

**Table 3:** Spearman's correlation coefficients (ρ) for key biochemical markers.

Part A. Low PTH group (hypoparathyroid pattern)							
Parameter	pcCa	Ionized Ca	P	Mg	ALP	Vitamin D	PTH
pcCa	1.000	0.825**	-0.314*	0.212	-0.205	-0.138	0.187
Ionized Ca	0.825**	1.000	-0.252	0.020	-0.082	-0.262*	0.265*
P	-0.314*	-0.252	1.000	-0.194	0.178	-0.060	-0.062
Mg	0.212	0.020	-0.194	1.000	0.275	-0.092	-0.054
ALP	-0.205	-0.082	0.178	0.275	1.000	-0.259	0.044
Vitamin D	-0.138	-0.262*	-0.060	-0.092	-0.259	1.000	-0.239
PTH	0.187	0.265*	-0.062	-0.054	0.044	-0.239	1.000

Part B. Normal/high PTH group (PTH-sufficient / secondary hyperparathyroid pattern)							
Parameter	pcCa	Ionized Ca	P	Mg	ALP	Vitamin D	PTH
pcCa	1.000	0.853**	-0.148	0.230**	0.081	0.089	-0.024
Ionized Ca	0.853**	1.000	-0.028	0.268**	0.025	-0.013	-0.093
P	-0.148	-0.028	1.000	0.117	-0.314**	-0.151	-0.108
Mg	0.230**	0.268**	0.117	1.000	0.047	0.032	0.048
ALP	0.081	0.025	-0.314**	0.047	1.000	-0.116	0.333**
Vitamin D	0.089	-0.013	-0.151	0.032	-0.116	1.000	0.005
PTH	-0.024	-0.093	-0.108	0.048	0.333**	0.005	1.000

*P < 0.05, **P < 0.01 (2-tailed Spearman’s ρ).

P, phosphorus; Mg, magnesium; ALP, alkaline phosphatase; PTH, parathyroid hormone; 25(OH)D, 25-hydroxyvitamin D. Pairwise deletion; N varies by correlation pair.

### 3.3 Biochemical profiles across etiological and age subgroups

When stratified by age (**Table 4A**), the severity of hypocalcemia was similar across neonates, infants, and children, with no significant differences in pcCa (1.40 ± 0.27 vs 1.35 ± 0.23 vs 1.44 ± 0.24 mmol/L; p=0.64) or ionized calcium (0.67 ± 0.15 vs 0.66 ± 0.13 vs 0.64 ± 0.13 mmol/L; p=0.638). In contrast, several accompanying biochemical parameters differed by age. Protein and albumin increased with age (protein: 58.3 [54.4–62.7] vs 56.8 [54.0–70.0] vs 75.1 [70.0–76.9] g/L; p<0.001; albumin: 36.2 ± 6.1 vs 37.1 ± 4.35 vs 41.8 ± 4.35 g/L; p=0.006). Phosphate was highest in neonates (2.91 ± 0.62 mmol/L) compared with infants and children (2.31 ± 0.69 and 2.43 ± 0.75 mmol/L; p=0.04). ALP peaked in infants and young children (757.3 [508.0–999.6] U/L), exceeding values in neonates and children (219.9 [144.5–302.3] and 271.1 [193.1–441.4] U/L; p<0.001). Vitamin D was lowest in infants and young children (18.5 [13.0–27.4] nmol/L) and higher in neonates and children (40.1 [26.1–56.9] and 49.8 [36.8–78.3] nmol/L; p<0.001). PTH also differed markedly by age, with the highest levels in infants and young children (248.0 [151–339] pg/mL; p<0.001). Magnesium and glucose did not vary significantly across age strata (p=0.11 and p=0.139, respectively).

**Table 4:** Key biochemical parameters stratified by age group and etiology.

Part A. Key biochemical parameters by age group				
Parameter	Neonates (mean ± SD) median (IQR)	Infants and young children (mean ± SD) median (IQR)	Children (mean ± SD) median (IQR)	P-value
pcCa (mmol/L)	1.40 ± 0.27	1.35 ± 0.23	1.44 ± 0.24	0.64
Ionized Ca (mmol/L)	0.67 ± 0.15	0.66 ± 0.13	0.64 ± 0.13	0.638
Protein (g/L)	58.3 (54.4-62.7)	56.8 (54.0-70.0)	75.1 (70.0-76.9)	<0.001
Albumin (g/L)	36.2 ± 6.1	37.1 ± 4.35	41.8 ± 4.35	0.006
P (mmol/L)	2.91 ± 0.62	2.31 ± 0.69	2.43 ± 0.75	0.04
Mg (mmol/L)	0.59 (0.51-0.67)	0.74 (0.66-0.78)	0.74 (0.69-0.81)	0.11
ALP (U/L)	219.9 (144.5-302.3)	757.3 (508.0-999.6)	271.1 (193.1-441.4)	<0.001
Vitamin D (nmol/L)	40.1 (26.1-56.9)	18.5 (13.0-27.4)	49.8 (36.8-78.3)	<0.001
PTH (pg/mL)	38.8 (19.5-88.2)	248.0 (151-339)	51.1 (11.2-263.7)	<0.001
Glucose (mmol/L)	4.0 (3.6-5.1)	5.1 (4.6-5.7)	5.4 (4.7-6.1)	0.139

Part B. Key biochemical parameters stratified by etiology				
Parameter	DiGeorge (N = 17)	DiGeorge+VDD (N = 10)	HypoPTH (N = 24)	HypoPTH+VDD (N = 18)
Age (days)	1683.35	782.0	1227.2	548.2
Ionized Ca (mmol/L)	0.62	0.65	0.57	0.67
Protein (g/L)	67.0	64.7	60.9	55.1
P (mmol/L)	2.9	2.6	2.7	2.9
ALP (U/L)	148.4	351.1	287.4	428.0
Vitamin D (nmol/L)	117.1	28.0	84.1	25.9
PTH (pg/mL)	6.3	11.4	22.2	25.2

Values shown as mean (normally distributed) or median (non-normally distributed). P-values by ANOVA or Kruskal–Wallis test, as appropriate.

**Figure 3:**
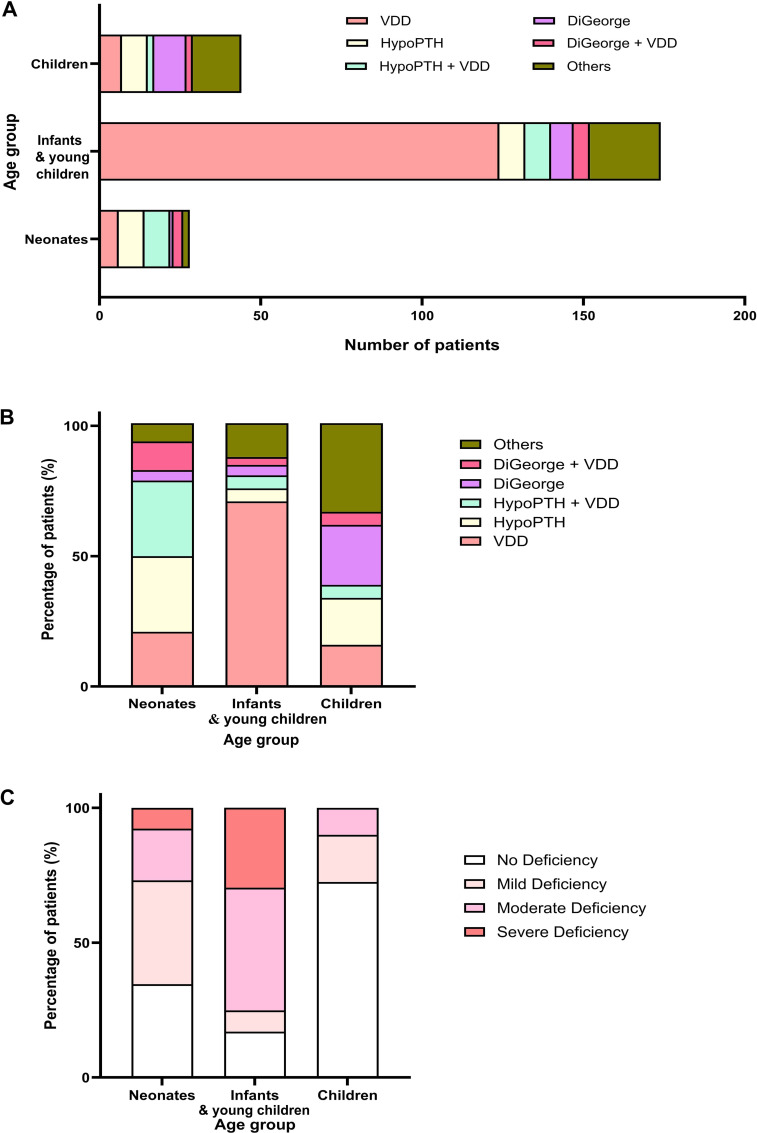
Age-stratified distribution of underlying etiologies and vitamin D status in 246 children with severe hypocalcemia. **(A)** Stacked horizontal bars display the absolute number of patients in each etiological category—vitamin D deficiency (VDD), isolated hypoparathyroidism (HypoPTH), HypoPTH + VDD, DiGeorge syndrome without VDD (DiGeorge), DiGeorge + VDD, and other/unknown causes—within three predefined age groups: neonates (1 day to 28 days), infants and young children (>28 days to 24 months), and children (>24 months). **(B)** Stacked vertical bars show the percentage contribution of each etiology to the total hypocalcemia burden in each age group. **(C)** Proportional bars illustrate the severity of vitamin D status in each age group, categorized as no deficiency, mild deficiency (30–49 nmol/L), moderate deficiency (12.5–29 nmol/L), or severe deficiency (<12.5 nmol/L). Infants and young children contributed the great majority of VDD cases, whereas congenital and mixed etiologies were relatively more frequent in neonates and older children. Severe and moderate VDD predominated in infants and young children, underscoring their heightened vulnerability.

Across etiological subgroups (**Table 4B**), age and key calcium–phosphate regulatory markers showed distinct distributions (**Figure 3**). The VDD group was youngest (age 236.1 days) and exhibited a profile characterized by higher PTH (261.7 pg/mL) and higher ALP (768.1 U/L) with the lowest vitamin D (18.4 nmol/L) (p<0.001 for age, ALP, vitamin D, and PTH). In contrast, DiGeorge and HypoPTH-spectrum groups showed suppressed PTH (DiGeorge 6.3 pg/mL; DiGeorge+VDD 11.4 pg/mL; HypoPTH 22.2 pg/mL; HypoPTH+VDD 25.2 pg/mL; p<0.001) alongside higher phosphate (approximately 2.6–2.9 mmol/L; p<0.001). Ionized calcium differed modestly across etiologies (p=0.032), with the lowest value observed in the HypoPTH group (0.57 mmol/L). The Others category showed the highest PTH (328.8 pg/mL) and ALP (854.1 U/L), with comparatively lower phosphate (1.7 mmol/L) (p<0.001).

Overall, these age- and etiology-stratified profiles demonstrate that while hypocalcemia severity was comparable across age groups, accompanying biochemical patterns clearly separated PTH-deficient states (low PTH with higher phosphate) from secondary hyperparathyroidism driven by vitamin D deficiency (high PTH with elevated ALP and low 25(OH)D).

### 3.4 Predictors of major clinical outcomes

#### 3.4.1 Seizures

Seizures were not only the most frequent presentation of hypocalcemia in this cohort, but their occurrence was also linked to several key factors. On univariate analysis (see **Table 5A**), children who experienced seizures were significantly younger (median age ~63 days) compared to those who did not have seizures (median 173 days; *p* = 0.002). Infants and young children dominated the seizure group (76% of those with seizures), whereas nearly half of non-seizure patients were older children. Seizure patients also had more severe biochemical abnormalities: the mean ionized calcium was 0.65 ± 0.12 mmol/L in the seizure group vs. 0.69 ± 0.15 mmol/L in non-seizure patients (*p* = 0.037), and their total calcium was lower as well (1.35 vs 1.46 mmol/L, *p* = 0.003). Notably, vitamin D status differed markedly between the groups – 80% of patients who had seizures were diagnosed with vitamin D deficiency, compared to 50% of those without seizures (*p* < 0.001). Consistent with this, the median 25(OH)D level among children presenting with seizures was significantly lower (21.0 nmol/L) than in those without seizures (47.1 nmol/L). Children presenting with seizures had modestly higher serum phosphate compared with those without seizures (mean 2.44 vs 2.18 mmol/L, *p* = 0.034). There were no differences in serum magnesium or albumin between the groups to suggest these as contributors, aside from a slightly lower protein level in seizure patients.

**Table 5:** Factors associated with seizures in children with severe hypocalcemia.

Part A. Univariate comparison of patients with and without seizures				
Characteristic / factor	Unit / category	No seizure (N = 51)	Seizure (N = 195)	P-value
Age	days	173 (60-2786)	63 (48-2871)	0.002
Body Weight	kg	6.5 (3.9-23.0)	4.5 (3.6-5.6)	0.007
Age Group	Newborn	8 (15.7%)	20 (10.3%)	<0.001˦
	Infant and young children	25 (49.0%)	149 (76.4%)	
	Children	18 (35.3%)	26 (13.3%)	
Vitamin D Deficiency*	Yes	25 (50.0%)	140 (80.0%)	<0.001^a^
	No	23 (46.0%)	43 (24.6%)	
pcCa	mmol/L	1.46 ± 0.29	1.35 ± 0.22	0.003
Ionized Ca	mmol/L	0.69 ± 0.15	0.65 ± 0.12	0.037
Protein	g/L	62 (56.4-70)	58.3 (53.6-66.2)	0.014
P	mmol/L	2.18 ± 0.82	2.44 ± 0.68	0.034
Vitamin D	nmol/L	47.1 (19.3-98.1)	21.0 (13.1-42.6)	<0.001

Part B. Multivariate logistic regression predictors for seizures				
Variable	Coefficient (B)	OR	95% CI	p-value
Ionized Calcium	-2.987	0.05	0.003 – 0.83	0.036
Vitamin D	-0.011	0.99	0.98 – 1.00	<0.001

Model diagnostics: Hosmer–Lemeshow χ² = 6.35 (p = 0.61). Values are median (IQR) or Fmean ± SD, depending on normality (Shapiro–Wilk test). ^a^χ² test; other p-values by Mann−Whitney U or independent−samples t−test as appropriate.

**Figure 4:**
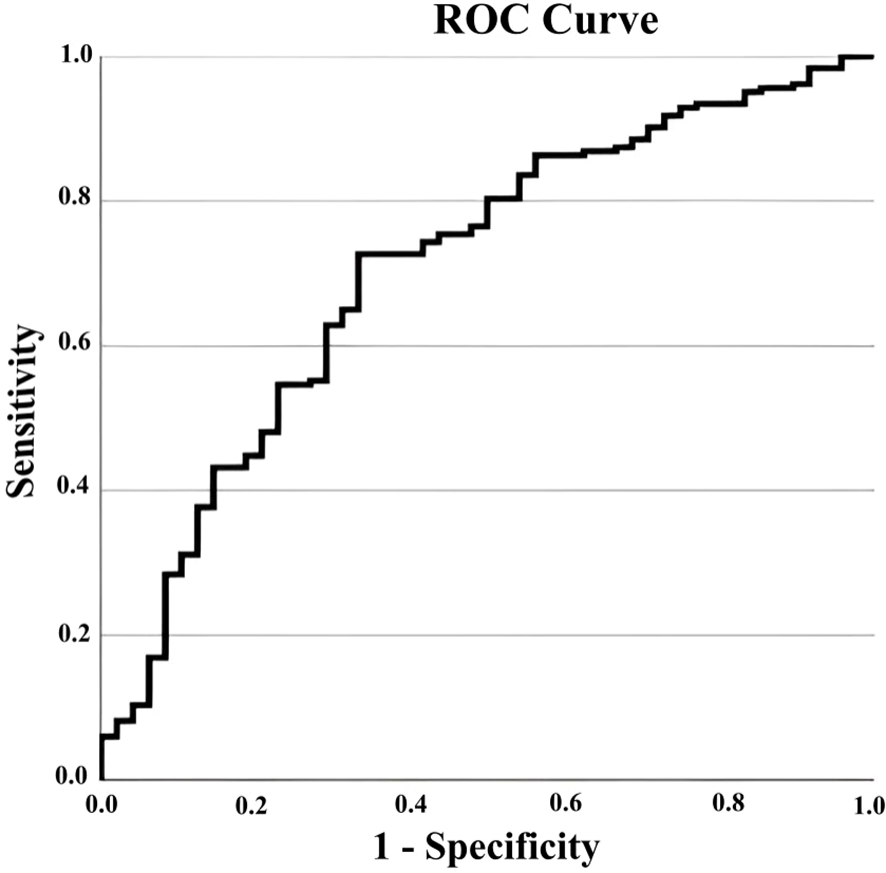
ROC curve for the seizure prediction model in pediatric hypocalcemia Receiver operating characteristic (ROC) curve assessing the discriminatory performance of the multivariable logistic regression model for seizure risk. The model incorporates ionized calcium and 25-hydroxyvitamin D concentrations as predictors. The area under the curve (AUC = 0.706; 95% CI: 0.63–0.78) indicates acceptable discriminatory capacity. The curve validates the independent predictive value of both hypocalcemia and vitamin D deficiency in identifying children at highest risk for neurologic complications.

In a multivariate logistic regression analysis (**Table 5B**), two variables emerged as significant independent predictors of seizure occurrence, after adjusting for potential confounders. Lower ionized calcium was associated with higher odds of seizures (OR ~0.05 per +1 mmol/L change in ionized Ca; *p* = 0.036). In addition, lower 25(OH)D levels independently predicted seizures (OR ~0.989 per +1 nmol/L, *p* < 0.001. Younger age showed a borderline association (OR ~0.999 per day older, *p* = 0.073), consistent with the vulnerability of young infants to hypocalcemic seizures, though this did not reach strict significance in the model. The regression model had a fair discriminative ability (area under ROC curve 0.706, *p* < 0.001) and was well-calibrated to the data (Hosmer–Lemeshow test *p* = 0.61) (**Figure 4**). In summary, these findings underscore that the depth of hypocalcemia and coexisting vitamin D deficiency are the key drivers of neurologic excitability in pediatric hypocalcemia, with age as an additional consideration.

#### 3.4.2 Cardiac events

Cardiac events related to hypocalcemia were observed in 14 out of 246 children (5.7%). As shown in **Table 6A**, these patients were significantly younger (median age 63 days vs. 173 days, *p* < 0.001) and had lower body weight (median 4.5 kg vs. 6.5 kg, *p* < 0.001) compared to those without cardiac manifestations. Congenital heart disease was more frequent in the cardiac involvement group (50% vs. 23.3%, *p* = 0.049). Biochemically, children with cardiac events had significantly lower serum protein and vitamin D levels (median 21.0 nmol/L vs. 47.1 nmol/L, *p* < 0.001). Thus, in univariate comparisons, younger age, lower weight, coexisting CHD, lower protein, and especially lower vitamin D levels were associated with increased risk of cardiac complications in hypocalcemic children. However, when these variables were entered into a multivariate logistic regression model (**Table 6B**), none emerged as a statistically significant independent predictor of cardiac involvement. The multivariate model was likely underpowered to detect independent predictors due to the small number of cardiac events (14 cases) in our study.

**Table 6:** Factors associated with cardiac involvement in children with severe hypocalcemia.

Part A. Univariate comparison				
Characteristic / factor	Category / unit	No cardiac involvement (N = 232)	Cardiac involvement (N = 14)	P-value
Age	days	173 (60-2786)	63 (48-2871)	<0.001
Body weight	kg	6.5 (3.9-23.0)	4.5 (3.6-5.6)	<0.001
CHD	Yes	54	7	0.049
	No	141	4	
	N/A	37	3	
Protein	g/L	60.9 (56.0-67.0)	54.8 (47.0-60.0)	0.009
Vitamin D	nmol/L	47.1 (19.3-98.1)	21.0 (13.1-42.6)	<0.001

Part B. Multivariate logistic regression predictors for cardiac involvement				
Variable	OR	95% CI (Lower bound)	95% CI (Upper bound)	P-value
Age	1.001	0.997	1.005	0.619
Body Weight	0.781	0.459	1.429	0.364
Protein	0.922	0.846	1.005	0.069
Vitamin D	0.989	0.782	1.251	0.332
CHD	1.747	0.686	4.45	0.242

CHD, congenital heart disease.

Values are median (IQR) unless stated otherwise. p-values from Mann−Whitney U (continuous) or χ²/Fisher’s exact test (categorical). None of the variables retained statistical significance in multivariate analysis.

**Figure 5:**
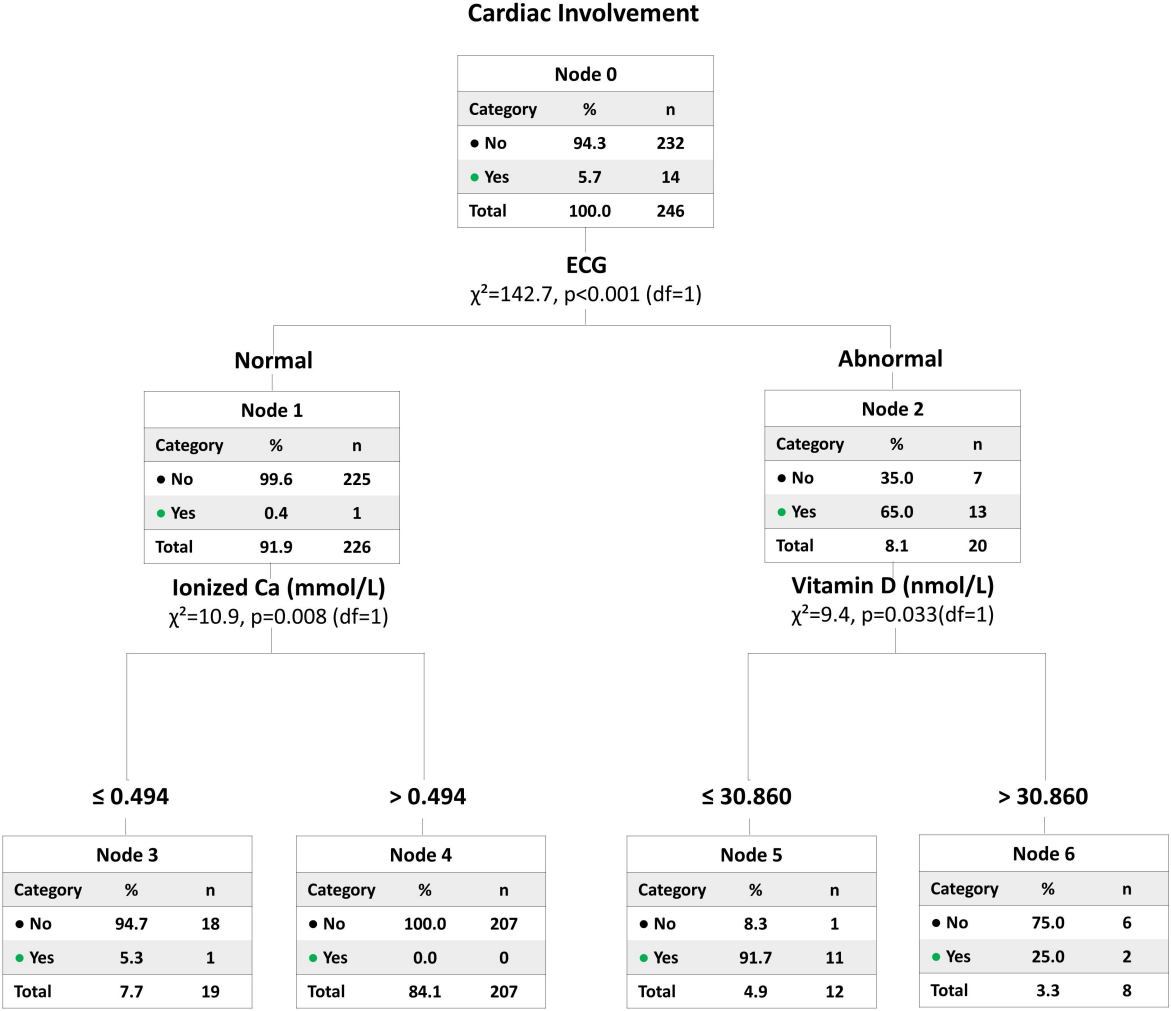
Decision tree identifying risk patterns for cardiac involvement. A classification-tree analysis was performed to stratify the risk of cardiac involvement (e.g., cardiogenic shock, heart failure) based on electrocardiogram (ECG) findings, ionized calcium levels, and 25-hydroxyvitamin D status. The presence of ECG abnormalities was the strongest predictor, conferring a 65.0% risk, which escalated to 91.7% in patients with concurrent lower 25-hydroxyvitamin D levels (≤ 30.86 nmol/L). Among patients with normal ECGs, profound ionized calcium depletion (≤ 0.494 mmol/L) defined a residual-risk subgroup (5.3%). The tree illustrates the hierarchical and multifactorial nature of hypocalcemia-induced cardiac complications and may assist in clinical triage.

To further clarify how combinations of clinical and laboratory variables might classify children at risk for cardiac involvement, we performed a decision tree analysis (**Figure 5**). The model first separated patients based on ECG findings: those with abnormal ECGs had a substantially higher risk of documented cardiac involvement (65% vs. 0.4% in those with normal ECGs). Among those with abnormal ECGs, low vitamin D (≤ 30.9 nmol/L) further increased the likelihood of cardiac events. In patients with normal ECGs, a very low ionized calcium (≤0.494 mmol/L) was more common among those with cardiac symptoms. However, despite these observed patterns, no single variable or split in the tree was sufficient to classify all cases with statistical certainty.

### 3.5 Length of hospital stay by etiology

**Figure 6:**
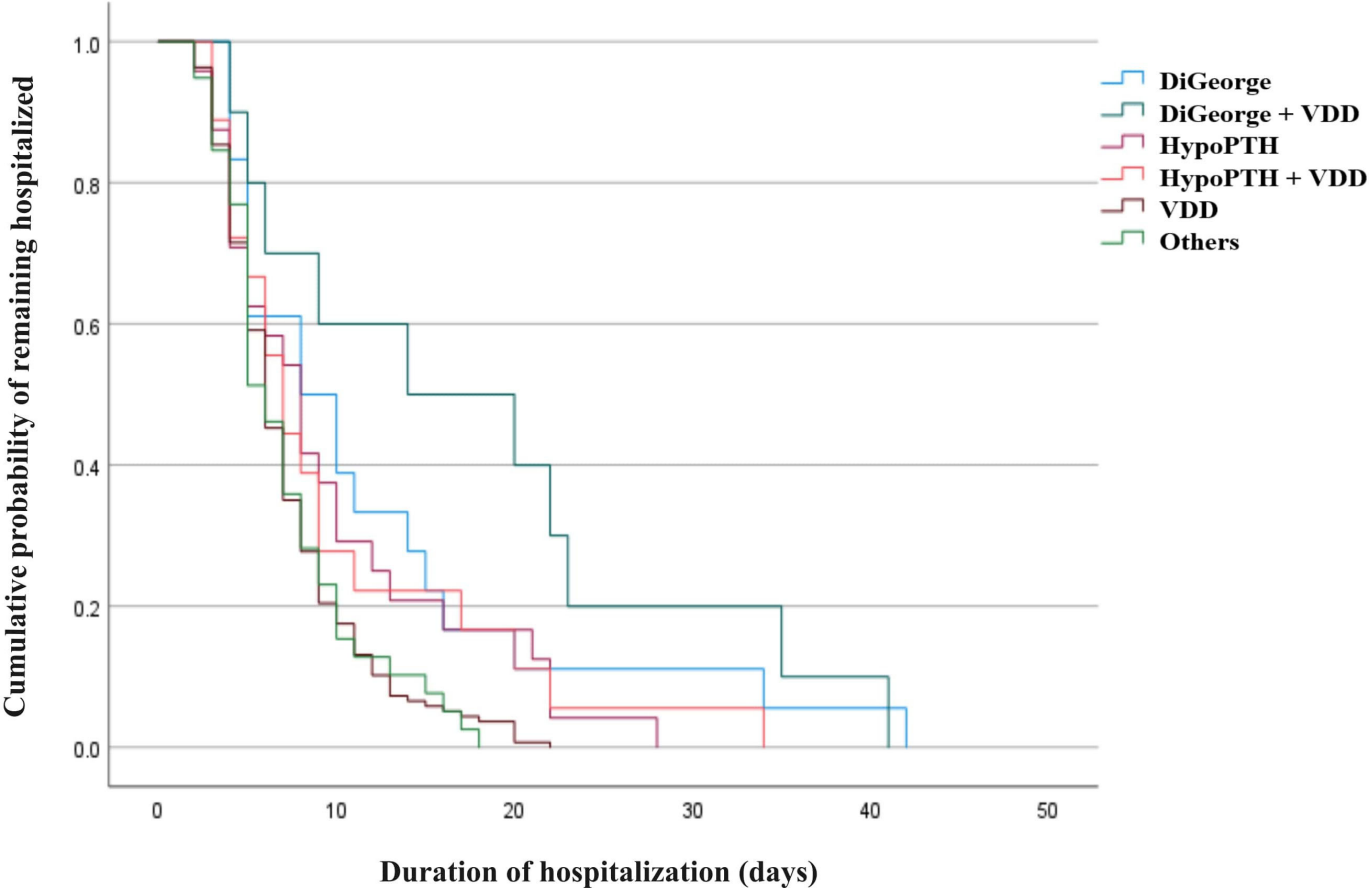
Kaplan-Meier curves for length of hospital stay by etiological group. The plot displays the cumulative proportion of patients remaining in the hospital over time (in days), stratified by the primary etiological diagnosis. The curves illustrate the differences in the duration of hospitalization among the groups, with patients with DiGeorge syndrome showing the longest stays and those with isolated Vitamin D deficiency showing the shortest.

The duration of hospital stays varied according to the underlying cause of hypocalcemia, highlighting differences in illness severity and management complexity. A Kaplan–Meier analysis of time to discharge (**Figure 6**) demonstrated that children with congenital forms of hypocalcemia had more prolonged admissions than those with nutritional causes. Patients with DiGeorge syndrome (22q11.2 deletion) – especially when compounded by vitamin D deficiency – had the longest median hospital stays, often exceeding one week. Those with isolated hypoparathyroidism (without DiGeorge) had intermediate-length hospitalizations. In contrast, children with straightforward nutritional vitamin D deficiency (with no other comorbidity) and those classified as “Other” minor causes were discharged the shortest time, often after only a few days of therapy. Statistical comparison across etiological subgroups confirmed significant differences in length of stay (p<0.001 by log-rank test). These patterns suggest that underlying etiology is a key determinant of resource utilization. . .

Cox regression analysis (**Table 7**) confirmed that the underlying cause of hypocalcemia is an independent predictor of hospital length of stay (Omnibus χ² = 16.9, p < 0.001). Patients with nutritional vitamin D deficiency were generally discharged sooner, while those with congenital or complex etiologies - such as DiGeorge syndrome or combined with hypoparathyroidism and vitamin D deficiency - experienced significantly longer admissions. The hazard ratio for cause was 1.23 (95% CI: 1.11–1.36), indicating a substantial effect of etiology on discharge rates. These findings reinforce that early identification of etiology is critical for anticipating resource utilization and guiding clinical management in pediatric hypocalcemia.

**Table 7:** Cox proportional−hazards analysis of time−to−discharge by etiologic group.

Etiology	P-value	Hazard ratio (Exp(B))	95% CI for Exp(B)
VDD^a^	—	1.00	—
DiGeorge	0.008	0.59	0.41 – 0.86
DiGeorge + VDD	0.092	0.69	0.45 – 1.06
HypoPTH	0.073	0.75	0.54 – 1.03
HypoPTH + VDD	0.020	0.66	0.47 – 0.92
Others	0.355	0.89	0.69 – 1.15

VDD, vitamin D deficiency; HypoPTH, hypoparathyroidism.

a Reference category. HR < 1 indicates a slower discharge rate (longer length of stay) relative to the reference group.

## 4 Discussion

In this retrospective cohort of pediatric hypocalcemia, nutritional vitamin D deficiency emerged as the most common etiology, particularly in infants and young children. Over two-thirds of our patients had low 25(OH)D levels, a burden of hypovitaminosis D consistent with reports from Vietnam and neighboring regions (15, 16, 29). Our finding that infants and young children accounted for many severe cases aligns with global observations that the most vulnerable age for symptomatic hypocalcemia is in the first months of life. A U.K. surveillance study reported an annual incidence of approximately 3.5 per million children for hypocalcemic seizures due to hypocalcemia, with infants (especially those of Asian or African descent) comprising most cases (30). Even in high-resource countries, current preventive strategies have not fully averted such life-threatening manifestations of vitamin D deficiency. Although maternal 25-hydroxyvitamin D data were available for only a small subset, the prevalence of maternal vitamin D insufficiency was high (90%). This figure mirrors the high prevalence of hypovitaminosis D previously reported among Vietnamese women of reproductive age (15). These preliminary data therefore identify maternal vitamin D deficiency as an upstream determinant of early-life hypocalcemia and underscore the need to extend preventive strategies beyond infancy.

Our study also demonstrated that biochemical profiles differed markly by etiology, offering diagnostic insight. In vitamin D deficiency, we observed the expected hallmarks of secondary hyperparathyroidism: low 25(OH)D, elevated PTH, high ALP, and often low-normal phosphorus. In contrast, patients with hypoparathyroidism showed inappropriately low PTH levels despite profound hypocalcemia, along with hyperphosphatemia and relatively lower ALP, consistent with absent PTH activity. Cases of combined etiologies (DiGeorge syndrome plus vitamin D deficiency) displayed intermediate biochemical patterns - a blunted PTH response that was higher than in pure hypoparathyroidism but lower than expected for the degree of hypocalcemia in nutritional vitamin D deficiency. These distinct laboratory signatures are in line with established diagnostic algorithms for pediatric hypocalcemia (1). The ability to stratify etiology by initial labs has important treatment implications - it allows timely initiation of the right therapy and appropriate anticipatory guidance for each condition.

Clinically, neuromuscular irritability was the hallmark of presentation. Seizures occurred in 79% of patients, often as the initial symptom. This mirrors the well-known propensity of hypocalcemia to provoke tetany and afebrile seizures in infants, who cannot verbalize milder symptoms (12). These findings make physiologic sense; infants have limited calcium reserves and immature parathyroid responses, so profound hypocalcemia, especially when compounded by vitamin D deficiency, readily triggers neuronal excitability (31). In line with this, 80% of seizure-affected children in our study had vitamin D deficiency, compared to about half of those without seizures, with markedly lower median 25(OH)D levels in the seizure group (21 nmol/L vs 47 nmol/L). Our experience accords with prior reports that many infants with vitamin D deficiency–related hypocalcemia present with convulsions (32). Collectively, these data reinforce vitamin D’s pivotal role in neuromuscular stability.

Cardiac manifestations, while relatively uncommon in our cohort (5.7% of patients), underscore the potential gravity of extreme hypocalcemia. Affected children exhibited features such as heart failure or cardiogenic shock, echoing previous descriptions of hypocalcemic cardiomyopathy and life-threatening arrhythmias in children (33). This clinical picture is well-documented, with numerous recent case series and reports detailing how profound, often vitamin D-dependent hypocalcemia can lead to severe but reversible dilated cardiomyopathy in infants and children (34, 35). On univariate analysis, those with cardiac involvement tended to be younger and of lower weight, more often vitamin D deficient, and had a higher prevalence of underlying congenital heart disease, suggesting that these factors may predispose to cardiovascular complications. However, in multivariate models, no single factor besides the severity of hypocalcemia itself emerged as an independent predictor of cardiac involvement. This indicates a multifactorial pathogenesis - the risk likely amplifies when hypocalcemia is superimposed on fragile cardiac conditions or profound vitamin D deficiency, but the degree of calcium lowering is the dominant determinant. A classification tree in our analysis similarly suggested that an abnormal ECG and extremely low calcium level together flagged most at-risk patients. Clinically, these findings highlight that all children with severe hypocalcemia merit careful cardiac monitoring, as predicting which patient will develop arrhythmia is difficult.

It is worth noting that the prolonged hospital stays observed in our infant subgroup, while not widely quantified in the literature, are plausible given the severity of disease. Children with congenital etiologies, notably DiGeorge syndrome (22q11.2 deletion) - especially when accompanied by vitamin D deficiency - experienced longer admissions compared to those with isolated nutritional deficiencies. These prolonged stays reflect the complexity inherent in managing syndromic conditions, as well as comprehensive assessments for associated anomalies (36, 37). Similarly, combined hypoparathyroidism and vitamin D deficiency cases required extended hospitalization due to compounded metabolic derangements. Conversely, patients with isolated vitamin D deficiency typically had shorter hospitalizations, reflecting the relative simplicity and rapid reversibility of nutritional etiologies through calcium and vitamin D supplementation (31, 38). Our Cox regression analysis supports these observations, identifying DiGeorge syndrome and combined hypoparathyroidism with vitamin D deficiency as independent predictors of prolonged hospital stay. These results emphasize the necessity for early and precise etiological classification, enabling clinicians to optimize resource allocation and predict the clinical course more accurately (12, 39). Public health efforts directed towards the prevention of vitamin D deficiency could substantially reduce hospitalization burden and healthcare costs associated with pediatric hypocalcemia.

Our findings have important public health and clinical implications. The predominance of vitamin D deficiency suggests that much of severe pediatric hypocalcemia in our setting is preventable, supporting stronger implementation of routine infant vitamin D supplementation and practical guidance on safe sun exposure. In Vietnam and similar Asian contexts, adherence may be limited by low sun exposure practices and low dietary vitamin D intake. Prevention should prioritize pregnant/breastfeeding women and young infants, especially exclusively breastfed infants without supplementation and those born to vitamin D–deficient mothers. Although maternal 25(OH)D was available only in a small subset, the very high deficiency rate among those tested supports this link. Clinically, early recognition of infants with profound hypocalcemia and low 25(OH)D may help prevent severe complications. Prospective studies with standardized documentation of feeding and supplementation are needed to refine risk stratification and guide scalable prevention strategies.

## 5 Strengths and limitations

The strengths of our study include the large sample size (to our knowledge, one of the largest cohorts of severe pediatric hypocalcemia reported) and the comprehensive data on both clinical outcomes and biochemical parameters. We also introduced predictive modeling (logistic regression and decision tree) to identify risk factors for complications, which is a novel approach in this field and could inform risk stratification tools for clinicians. Moreover, our setting in a developing country provides data that complement prior studies mostly from developed countries, thereby broadening the global understanding of pediatric hypocalcemia.

While this study provides meaningful insights into severe pediatric hypocalcemia in a real-world setting, certain limitations should be considered when interpreting the findings. First, the retrospective design relies on documentation quality and limits control over measurement timing. Pre-treatment sampling could not be guaranteed in all cases, potentially affecting biochemical values. In addition, samples were not uniformly fasting morning samples, which may have contributed to variability in serum phosphate measurements. Second, dietary calcium intake and supplementation history (vitamin D and calcium) could not be reliably quantified because caregivers frequently could not recall the exact product, dose, frequency, or duration; therefore, these variables were incomplete and were not suitable for dose–response analyses. Accordingly, supplementation exposure could only be summarized as yes/no/unknown when recorded, and detailed dose–response analyses were not feasible. Third, maternal 25(OH)D was available only for a small subset because maternal testing required additional consent and was often declined; consequently, maternal findings are descriptive and may be influenced by selection bias. Finally, as this cohort reflects real-world practice within a limited healthcare setting, generalizability to all Vietnamese children or other populations may be restricted, and prospective multicenter studies with standardized dietary/supplement documentation and uniform diagnostic workflows are needed.

## 6 Conclusion

Severe hypocalcemia in Vietnamese children was associated with substantial acute morbidity, most notably seizures and clinically significant cardiac complications. The etiologic spectrum was dominated by nutritional vitamin D deficiency, while PTH-deficient disorders (including hypoparathyroidism and DiGeorge-related hypoparathyroidism) represented an important minority with distinct biochemical signatures (notably higher phosphate and inappropriately low PTH). Age- and etiology-stratified analyses demonstrated that, although the absolute severity of hypocalcemia was similar across age groups, accompanying markers (PTH, ALP, phosphate, and 25(OH)D clearly separated PTH-deficient states from secondary hyperparathyroidism associated with nutritional deficiency.

These findings emphasize the need for earlier recognition and targeted evaluation of severe hypocalcemia using a physiology-based approach and support strengthened preventive strategies for vitamin D deficiency in infants and mothers. Given the retrospective design and incomplete data on calcium intake and supplementation, prospective studies with standardized documentation of feeding, supplementation, and maternal status are warranted to refine risk stratification and optimize prevention and management pathways.

## Data Availability

The raw data supporting the conclusions of this article will be made available by the authors, without undue reservation.
